# Factors associated with severe occupational injuries at mining company in Zimbabwe, 2010: a cross-sectional study

**DOI:** 10.11604/pamj.2013.14.5.1148

**Published:** 2013-01-03

**Authors:** Chipo Chimamise, Notion Tafara Gombe, Mufuta Tshimanga, Addmore Chadambuka, Gerald Shambira, Anderson Chimusoro

**Affiliations:** 1 Department of Community Medicine, University of Zimbabwe, Zimbabwe; 2 Provincial Medical Directorate, Midlands Province, Zimbabwe

**Keywords:** Severe occupational injuries, mine workers, Zimbabwe

## Abstract

**Introduction:**

Injury rate among mining workers in Zimbabwe was 789/1000 workers in 2008. The proportion of severe occupational injuries increased from 18% in 2008 to 37% in 2009. We investigated factors associated with severe injuries at the mine.

**Methods:**

An unmatched 1:1 case-control study was carried out at the mine, a case was any worker who suffered severe occupational injury at the mine and was treated at the mine or district hospital from January 2008 to April 2010, a control was any worker who did not suffer occupational injury during same period. We randomly selected 156 cases and 156 controls and used interviewer administered questionnaires to collect data from participants.

**Results:**

Majority of cases, 155(99.4%) and of controls 142(91%) were male, 127(81.4%) of cases and 48(30.8%) of controls worked underground. Majority (73.1%) of severe occupational injuries occurred during night shift. Underground temperatures reached 500C. Factors independently associated with getting severe occupational injuries included working underground (AOR = 10.55; CI 5.97-18.65), having targets per shift (AOR = 12.60; CI 3.46-45.84), inadequate PPE (AOR= 3.65 CI 1.34-9.89) and working more than 8 hours per shift (AOR = 8.65 CI 2.99-25.02).

**Conclusion:**

Having targets exerts pressure to perform on workers. Prolonged working periods decrease workers’ attention and concentration resulting in increased risk to severe injuries as workers become exhausted, lose focus and alertness. Underground work environment had environmental hazards so managers to install adequate ventilation and provide adequate PPE. Management agreed to standardize shifts to eight hours and workers in some departments have been supplied with adequate PPE.

## Introduction

Occupational injuries present a major public health problem resulting in serious social and economic consequences that could be prevented if appropriate measures are taken. Majority of world′s workforce does not have access to occupational health services [1]. Estimated economic loss caused by work-related injuries and disease is equivalent to 4% of the world′s gross national product [2]. The impact is 10 to 20 times higher in developing countries [3]. According to Leigh, 100 million occupational injuries occur throughout the world each year [4].

National Institute of Occupational Safety and Health (NIOSH) USA estimates that at least 10,000,000 persons suffer injuries on job each year. About 30% of these injuries are severe. In Zimbabwe occupational injuries are among the top ten health priorities [5]. The highest numbers of occupational injuries in Zimbabwe occur in the construction, mining, and manufacturing industries. The injury rate among mining workers in Zimbabwe was 131 per 1000 exposed workers per year as of 1998 [6]. This figure rose to 789/1000 workers in 2008 [7]. A survey of 1585 informal/small scale workers in rural and urban Zimbabwe found occupational injury and mortality rates similar to those found in large scale/formal sector [8].

The mining company under study is a multiple mineral extracting company which was established in 1906. Since then it has transformed into one of the country′s largest mining companies with more than 3 000 employees in one of their divisions who are exposed to continuous potential risk of occupational injuries. The mine operates underground and open cast mines.

The proportion of severe occupational injuries at the mine increased from 18% in 2008 to 37% in 2009. The proportion is very high as compared to the maximum reported by NIOSH of 30% and the 21.9% recommended by ILO [9]. Factors contributing to severe occupational injuries were not clear to mine management; therefore we investigated factors associated with severe occupational injuries at the mine. Specifically we assessed personal, administrative, engineering factors associated with occupational injuries and the availability and use of personal protective equipment (PPE) at the mine form 2008 to 2010.

## Methods

A 1:1 unmatched case-control study was carried out at the mine, with all mine workers as our study population. A case was any worker who suffered a severe occupational injury at the mine and was treated at the mine hospital during the period January 2008-April 2010. A control was any worker who did not suffer an occupational injury during the same period. We included any mine worker who suffered severe occupational injury at the mine and was treated at the mine hospital or the district hospital from January 2008 to April 2010 but excluded any mine worker who suffered an occupational injury at the mine but was not treated at the mine hospital or the district hospital from January 2008 to April 2010.

Using StatCalc function of EPI Info Version 3.5.1 of 2008 and calculating the sample size at 95% confidence level, 80% power and expected occupational injury rate of 25% among mine workers and using the variable of not having received pre-employment training, OR = 2.04; CI = 1.50-2.77, [10] a minimum sample size of 156 cases and 156 controls was calculated. A sampling frame was prepared from cases from the district hospital outpatients register and occupational injuries register at the mine. From the sampling frame, cases were selected through the process of simple random selection. Random numbers were generated using random number function of a scientific calculator and cases with corresponding numbers were selected into the study. Controls were randomly selected from the workers of the mine through the same procedure as for cases and using the employee register as the sampling frame.

A conceptual Framework PRECEDE and PROCEED Model was used to design the questionnaire to assess factors associated with severe occupational injuries at the mine. The questionnaire was developed using constructs in the framework which include: Predisposing, Enabling and Reinforcing factors. Two focus group discussions one with twenty eight people and the other with thirty three people were held to identify thematic areas which helped in designing the questionnaire for study participants. A pre-tested interviewer administered questionnaire was used to collect information from study participants. The questionnaire was pre tested at a different division of the mining company for validity. Medical records were reviewed to assess case classification and management.

A check list was used to assess PPE availability and use, availability of occupational safety policies and regulations, safety warning signs and maintenance of equipment. Mine management and occupational health officer were interviewed as key informants for information on administrative and engineering factors.

Actual measurement of temperatures, illumination and noise levels was performed to assess environmental stresses. Statistical analysis of quantitative data was done using Epi info 3.5.1 Permission to carry out research was obtained from Mine Management, Provincial Medical Director Midlands Province, and Health Studies Office. Informed written consent was obtained from participants and confidentiality was maintained throughout the study. Ethical clearance was obtained from Medical Research Council of Zimbabwe.

## Results

We enrolled 156 cases and 156 controls for the study. The majority of study participants 155(99.4%) of cases and 142(91%) of controls were male, 127(81.4%) of cases and 48(30.8%) of controls worked underground and 127(81.4%) of cases and 17(11%) of controls had a maximum of primary school education. The median years in service was 13 (Q1 = 5 Q3 = 21) for cases and19 (Q1 = 11 Q3 = 26.5) for controls and median age was 37 (Q1 = 28 Q3 = 47) for cases and 41 (Q1 = 35 Q3 = 49.5) for controls (Table 1).

**Table 1:** Demographic Characteristics of Cases and Controls at a Mining Company, 2010

Variable	Cases	Controls	p-value
	n= 156(%)	n= 156(%)	
**Sex**			
Male	155(99.4)	142(91)	
Female	1(0.6)	14(9)	0.0005
**Country of origin**			
Outside	24	6	0.0002
Zimbabwe	132	150	
**Marital status**			
Married	97(62.2)	133(85.3)	0.0000
Not married	59(37.8)	23(14.7)	
**Department of work**			
Underground	127(81.4)	48(30.8)	0.0000
Open cast	29(18.6)	108(69.2)	
**Educational level**			
Primary	127(81.4)	17(11)	0.0000
Secondary	29(18.6)	138(89)	
**Median years in service**	13 (Q_1_=5 Q_3_= 21)	19 (Q_1_=11 Q_3_=26,5)	
**Median age**	37 (Q_1_=28 Q_3_= 47)	41 (Q_1_=35 Q_3_= 49.5)	

The majority of cases 111 (71.1%) had severe injuries occurring on the arms, followed by leg injuries at 26.3% (41 workers), injuries on the head accounted for 9% (14 workers) and injuries on the trunk only contributed 0.6% (1worker) of all severe injuries. The majority, 114 (73.1%) of severe occupational injuries occurred while workers were on night shift.

On analysis of personal risk factors, we found that male miners were 15.3 times more likely to be severely injured than female miners; mine workers who had sleep problems were 9 times more likely to get severely injured than those who did not have and those who reported dissatisfaction with their work were 20 times more likely to get severely injured than those who were satisfied with their jobs.

Workers who worked more than eight hours per shift were 14.5 times more likely to get severely injured than those who worked eight or less hours per shift, those who had targets to meet had a 43.4 times more risk of getting severely injured than those who did not have targets set for them. Workers who were not trained in the use of the equipment they were using were 5.2 times more likely to get severely injured than those who had prior training. Those who worked underground were 9.8 times more likely to get severely injured than those who worked on the surface. On engineering factors, we found that mine workers who were using manual equipment were 10.2 times more likely to get severe injuries than those who used automatic equipment; those who reported that their equipment did not suit their needs were 18.6 times more likely to get severe occupational injuries than those who fitted well with their machines/equipment. On personal protective equipment (PPE) factors, we found that workers who had not been trained in correct use of their PPE were 2.8 times more likely to get severe occupational injuries than those who were trained, those who did not use PPE correctly were 3.8 times more likely to get severely injured than those who used it correctly and those who did not consistently use PPE while at work were 7.5 times more likely to be severely injured than those who consistently used PPE.

On multivariate analysis using stepwise logistic regression, working underground (AOR = 10.55; CI 5.97-18.65), having targets to achieve per work shift (AOR = 12.60; CI 3.46-45.84), inadequate PPE (AOR= 3.65; CI 1.34-9.89), working more than 8 hours per shift (AOR = 8.65; CI 2.99-25.02), using automatic equipment (AOR = 0.33; CI 0.13-0.81) and having at least secondary education (AOR = 0.08; CI 0.03-0.18) remained independently associated with experiencing severe occupational injuries at the mine (Table 2).

**Table 2:** Factors Associated with Severe Occupational Injuries at a Mining Company in Zimbabwe, 2010

Variable		Severe Injury		OR	95% CI	AOR	95% CI
		Yes	No				
Working underground	Yes	127	48	9.85	5.81-16.70	10.55	5.97 - 18.65
	No	29	108				
Having targets to achieve per work shift	Yes	151	64	43.41	26.85- 65.84	12.60	3.46 - 45.84
	No	5	92				
Inadequate PPE	Yes	132	126	1.31	0.70-2.46	3.65	1.34 - 9.89
	No	24	30				
Working more than 8hours per shift	Yes	121	30	14.52	8.39-25.11	8.65	2.99 - 25.02
	No	35	126				
Using automatic equipment	Yes	153	69	10.24		0.33	0.13 - 0.81
	No	3	87				
Having secondary education	Yes	127	48	35.8	18.78-68.26	0.08	0.03 - 0.18
	No	29	139				

In all surface departments, Occupational Health and Safety policies were available and displayed in many places where workers could easily see and read. The same applied to danger warning signs. But steep slopes had no side rails and there were no guards on cutting edges and other sharp equipment except for the plant department. Ambient temperatures ranged from 29 to 29.8 degrees Celsius. The normal temperature for the day was 290C, noise levels were above recommended amounts of 85 dBA, ranging from 95 to 108 decibels, and illumination ranged from150 to 460 lux.

Underground, occupational health and safety policies were available at three workstations out of the six visited stations. There were no danger warning signs, no side rails to protect steep and slippery walkways and sharp edges were not guarded along all tunnels. Illumination was only provided by miners′ torches. Noise levels were from 150 up to 400 decibels during blasting and temperature was above 50°C in work areas where most of the activity was going on. The normal atmospheric temperature for the day was 28.6°C. Table 3 shows the environmental stressors and measurements observed against the threshold limit values (TLV).

**Table 3:** Threshold Limit Values for Environmental Stressors and Measurements Observed at the Mine, 2010

Work station	Illumination (TLV = 15-700 lux)	Noise level (TLV = 85 dBA)	Respirable dust (TLV= 2.5mg/m^3^)	Temperature (TLV = 25°C)	Guards on steep slopes	Danger warning signs
Surface Plant	150-460	95-108	4	29.8	Yes	Yes
Surface-loading bay	150-700	70-95	5	29	Yes	Yes
Surface Engineering	350	95-105	3	29	Yes	Yes
Underground		150-400	8	50	No	No

## Discussion

Having targets to achieve per shift was found to be associated with getting severely injured at the mine under study. Having targets set for a work shift exerts pressure to perform on workers. Targets make workers to focus on production and meeting the targets so much that they may disregard precautionary measures and put themselves at risk of being injured in the process. As production corresponds to remuneration, every effort would be directed towards achieving the targets so that workers get more money and short cuts may be taken, endangering workers. Findings from a study of transient risk factors for acute traumatic hand injury in China are consistent with our findings. They found that workers who were rushed to achieve targets were 15.5 times more likely to get injured than those who were not [11].

Working underground was significantly associated with getting severe occupational injuries. High temperatures and inadequate illumination negatively affects workers′ alertness leading to reduced concentration levels thereby increasing workers′ risk to severe occupational injuries. Lack of danger warning signs which keep reminding workers of areas which need extra precautions, steep slopes and slippery surfaces which had no side rails to support workers and prevent falls and sharp edges with no guards to prevent cuts, all contributed to making the underground work station a risky work area. Results of a study done in USA showed that occupational injury rate in underground mines was twice that of all other mining departments put together [12]. But contrary to our findings, a study by Hull et al showed that there was no difference in risk of occupational injuries by mine department [13].

Prolonged continuous working periods decrease workers′ attention and concentration levels resulting in increased risk to severe injuries.

Workers who work longer hours become very exhausted and lose focus and alertness due to tiredness. Results of a study done in USA on the impact of overtime and long working hours on occupational injuries and illnesses were consistent with our findings [14]. It was shown that working for more than 8 hours per shift resulted in a 61% increased risk for occupational injuries and that a strong dose response effect was observed where the longer the work shift resulted in increase in the risk of injuries [14].

Inadequate personal protective equipment and clothing was also significantly associated with getting severely injured at the mine. PPE puts a barrier between the worker and the hazard thereby protecting the worker from injury. Mining work is potentially risky and for a worker to work without adequate protective gear, is taking unnecessary risk. Our finding is consistent with findings from a study done in USA where results showed that 79% of the workers who got silicosis were putting on inadequate PPE in form of respirators [15].

Our study was not without limitations, fatalities were excluded from the study yet they could have been valuable information which could have brought in the healthy worker effect in our results. Privacy was limited as workers were interviewed at their work places and we did not have adequate time with workers as they wanted to achieve their targets.

## Conclusion

Factors independently associated with getting severely injured were working underground, having inadequate PPE, having targets to achieve per work shift and working more than 8 hours per shift. Underground work environment was not conducive to safe work performance due to high temperatures, unguarded steep slopes and unavailability of danger warning signs.

We recommended that management should set achievable targets for workers and offer performance awards to motivate production. Management should rotate workers through departments to lessen the period a workers are exposed to the risky underground workstation, provide adequate PPE and install adequate fans to ventilate underground work areas. The administrative controls to prevent occupational injuries should be strengthened so that workers adhere to the OSH policy and appropriate PPE be given as a last resort. In the long term, management should invest in engineering controls. Management should institute research to evaluate economic losses due to occupational injuries and Public Health Officers should investigate the injury investigation procedure at the mine to find ways of making it worker friendly and workers’ representative and mine managers to encourage workers to report accidents. Public Health Actions taken so far include: the management has agreed to standardize work shifts to 8 hours, to supply every worker with adequate PPE and workers in the loading bay and the plant had already been given their stipulated PPE in adequate amounts.
